# Not assessing the efficiency of multiple sequence alignment programs

**DOI:** 10.1186/1748-7188-9-18

**Published:** 2014-07-05

**Authors:** Andrew E Torda

**Affiliations:** 1 Centre for Bioinformatics, University of Hamburg, Bundesstr. 43, Hamburg 20146, Germany

**Keywords:** Plots, Units, Significant digits, Multiple sequence alignments

## Abstract

One can search for messages in the digits of π or a Kazakhstan telephone book, but there may be hidden messages closer to home. A recent publication in this journal purportedly compared a set of multiple sequence alignment programs. The real purpose of the article may have been to remind readers how to present scientific data.

## Main text

Multiple sequence alignments underpin many of our beliefs in biology ranging from the recognition of conserved sites to phylogeny. Fortunately, there are many freely available programs which tackle this task. Unfortunately, they can produce different results, so objective comparisons are invaluable to those using the software. Pais *et al.* presented such a comparison, but on available evidence, there was a second motive [1]. The paper very subtly demonstrates several aspects of data presentation, but is too discreet. It is left to the reader to extract the hidden treasures of data analysis and statistics.

**Figure 1:**
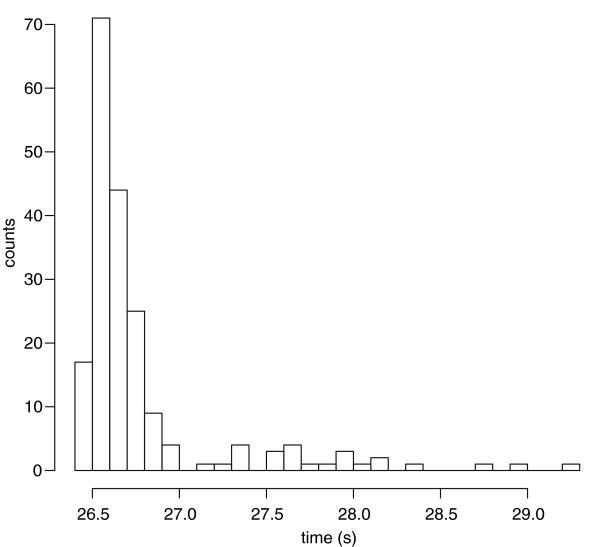
**Distribution of running times.** 195 repetitions were made as described in the text. Results are simple counts.

The authors ran nine multiple sequence alignment programs on a series of test sets. They collected estimates of computer time, memory usage and quality of the alignments. In each of these areas, their results are instructive. First, one can consider the estimates of computer time. The first item on the last row of their table makes several points. They quote "22 m 32.953 s". Given the convention that the uncertainty lies in the last digit, this would mean their measurement is meaningful up to one hundredth of a second. They know the time was 1323.95 s and not 1323.94 s. This could be a message from the authors, not to use so many decimal places, since we know that the timer resolution on a typical machine is at best 10^-2^ s. It may be that the authors quoted such an absurd number of decimal places to make a stronger point. They are saying that one could easily empirically estimate the uncertainty by running some of the calculations many times. This would quickly let one estimate the uncertainty and lead to a distribution as shown in Figure 1. This was run with one of the programs the authors used [2] and the data set they refer to as RV11 [3] and on a computer which was quiet, but not in single-user mode. Pais *et al.* must have wanted us to notice that, although the command line prints times to great accuracy, they do not have much meaning. It is difficult to say much more than the calculation in the figure takes about 26 ½ seconds. One can calculate the standard deviation (σ = 0.5 s) or variance, but it is not helpful with this kind of skewed distribution.

Pais *et al.* actually buried more meaning in their results and added a point about the correct use of units [1]. If one wants to discuss fruit supplies, you might say you have three apples and I have 30. You would not say you have three apples while I have two boxes plus six loose apples. In their table of timing results one is asked to compare values like "22 m 32.953 s" with "0 m 7.498 s" instead of 1353 s and 7.5 s. One is also reminded that the instructions to authors for Algorithms in Biology state that one should use SI units. The Bureau International des Poids et Mesures states that minutes are a non-SI unit, but should they be used, the abbreviation is min, not "m" and has been since 1948 [4].

**Figure 2:**
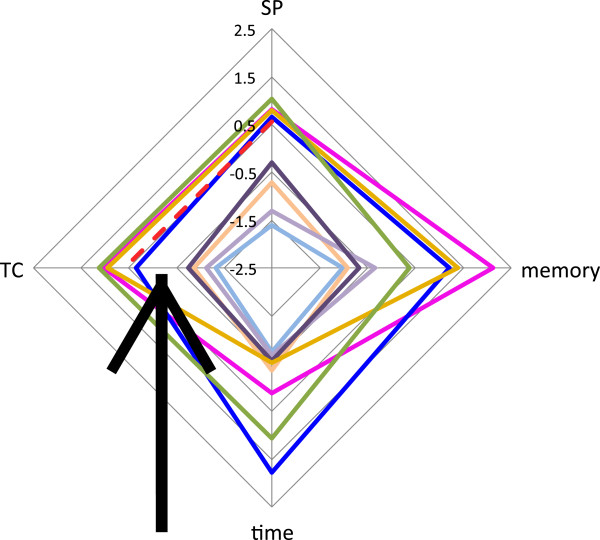
**A bad radar plot showing a non-Gaussian distribution.** The arrow marks the mean of the values on the axis. TC and SP are measures of alignment quality. Memory and time refer to memory usage and run time. The different coloured lines show the values for nine different programs. All values are dimensionless *z*-scores. Adapted from Pais *et al.*[1] without permission.

Pais *et al.*[1] also wanted to instruct the reader on the presentation of graphical data and simple statistics. To this end, one must remember the meaning of the *z*-score. This is simply a count of how many standard deviations an observation is from the mean. If we have Gaussian-distributed data, a *z-*score of 2 would quickly say that a point was within the extreme few percent of a distribution. In this study, the authors wanted to point out what an utterly meaningless and misleading transformation of data it can be when used wrongly. For a property such as computational time, the authors average the number from nine different programs. Would one expect these values to be Gaussian distributed? One can answer this by looking at Figure 2, extracted from Figure 1 of Pais *et al*. This shows nine quantities. For each quantity, the value from each program is converted to a *z*-score. The arrow on the figure marks the mean value where the points would be concentrated if the values were normally distributed. In fact, there is a dearth of points here. The data seems to be anti-Gaussian distributed or perhaps bi-modal. The data is not Gaussian-distributed on any of the axes or in any of the plots.

The authors hid even more meaning in order to make a point about radar plots and plots in general. If one wants to convey the idea of quality, one would construct plots so lower meant better or higher meant better or near to the centre was good or far from the centre was desirable. In this plot, values close to the centre are good for time and memory, but bad for alignment quality. This guarantees that a plot with the best possible program and the worst possible program must contain a tangle of crossed lines. This is what one sees on most of the plots in the paper. Continuing in this vein, the authors probably wanted to tell us just how much information can be lost with this kind of presentation. If you have a quantity such as time, a plot is normally scaled so the largest value fills the plot. A reader can look at the axis label and see if one is dealing with μs, s or 10^3^ s. If one converts to *z*-scores, one loses this. A reader can see that a point is one standard deviation from the mean, but does not know if the points span a range of nano-seconds or days.

A good article may not just present facts. It can also raise issues and pose questions. Pais *et al.* did not forget this when they say they used Friedmans and Dunns methods to assess statistical significance. Friedman worked in economics [5], where experiments are almost impossible to repeat and there is rarely enough data for bootstrapping or leave-one-out methods. One may wonder if his test is appropriate [6]. The authors prefer to refer to a proprietary program rather than the primary literature or even a text book, they did this to remind us to do so. Dunn explains that in his post test, "the null hypothesis to be tested is that the samples come from populations with identical, continuous distributions" [7]. It is left to the reader to decide whether this applies to discrepancies between multiple sequence alignment programs.

## Conclusions

Through the use of poorly presented numerical data, a sea of plots and inappropriate statistics the authors have offered a strong reminder of how to present data.

## Competing interests

The author has no interest in benchmarking multiple sequence alignment programs, no interest in making fun of others’ work and no other competing interests.
